# Intra-individual changes in DNA methylation not mediated by cell-type composition are correlated with aging during childhood

**DOI:** 10.1186/s13148-016-0277-3

**Published:** 2016-10-21

**Authors:** Kristina Gervin, Bettina Kulle Andreassen, Hanne Sagsveen Hjorthaug, Karin C. Lødrup Carlsen, Kai-Håkon Carlsen, Dag Erik Undlien, Robert Lyle, Monica Cheng Munthe-Kaas

**Affiliations:** 1 Department of Medical Genetics, Oslo University Hospital and University of Oslo, Oslo, Norway; 2 Department of Molecular Biology, Institute of Clinical Medicine, University of Oslo, Oslo, Norway; 3 Cancer Registry of Norway, Institute of population based Cancer Research, Oslo, Norway; 4 Department of Pediatrics, Oslo University Hospital and University of Oslo, Oslo, Norway; 5 School of Pharmacy, University of Oslo, Oslo, Norway; 6 Department of Pediatrics, Section of Hematology and Oncology, Oslo University Hospital, Oslo, Norway

**Keywords:** DNA methylation, Longitudinal, Aging, Cell-type composition, Childhood

## Abstract

**Background:**

Several studies have reported age-associated changes in DNA methylation in the first few years of life and in adult populations, but the extent of such changes during childhood is less well studied. The goals of this study were to investigate to what degree intra-individual changes in DNA methylation are associated with aging during childhood and dissect the methylation changes directly associated with aging from the effect mediated through variation in cell-type composition (CTC).

**Results:**

We performed reduced representation bisulfite sequencing (RRBS) in peripheral whole-blood samples collected at 2, 10, and 16 years of age. We identified age-associated longitudinal changes in DNA methylation at 346 CpGs in 178 genes. Analyses separating the effect mediated by CTC variability across age identified 26 CpGs located in 12 genes that associated directly with age. Hence, the CTC changes across age appear to act as a mediator of the observed DNA methylation associated with age. The results were replicated using EpiTYPER in a second sample set selected from the same cohort. Gene ontology analyses revealed enrichment of transcriptional regulation and developmental processes. Further, comparisons of the mean DNA methylation differences between the time points reveal greater differences between 2 to 10 years and 10 to 16 years, suggesting that the identified age-associated DNA methylation patterns manifests in early childhood.

**Conclusions:**

This study reveals insights into the epigenetic dynamics associated with aging early in life. Such information could ultimately provide clues and point towards molecular pathways that are susceptible to aging-related disease-associated epigenetic dysregulation.

**Electronic supplementary material:**

The online version of this article (doi:10.1186/s13148-016-0277-3) contains supplementary material, which is available to authorized users.

## Background

DNA methylation has diverse roles in many aspects of human biology (e.g., cell differentiation [1], genomic imprinting [2], and gene expression [3]). In mammals, DNA methylation is predominantly restricted to CpGs, which are unevenly distributed across the genome. DNA methylation is highly variable between individuals but also between cell types and tissues [4]. Variation in DNA methylation is also implicated in the development of diseases such as cancer [5], autoimmunity [6], and obesity [7].

For most cell types, DNA methylation patterns become set after cell differentiation and are generally considered relatively stable. However, DNA methylation also exhibits some dynamic characteristics, and DNA methylation landscapes change over a lifetime [8]. Age-associated changes in DNA methylation involve two distinct phenomena: epigenetic drift and the epigenetic clock [9], both involving intra-individual changes over time. However, while epigenetic drift [8] involves changes that are different between individuals, the epigenetic clock characterizes loci, which are systematic across individuals [10–12]. Changes in DNA methylation have been shown to occur during aging as well as during the development of age-related diseases, notably cancer [13], and are found to be gene-specific and genome-wide [14–19], tissue-specific [11, 20, 21], and tissue-independent [10, 21].

Genetics, the environment, and stochastic events are believed to influence epigenetic changes, which are likely to play important roles in mediating the risk for developing age-related diseases. However, a better understanding of the normal range of temporal DNA methylation changes is needed in order to fully investigate and interpret disease-associated epigenetic changes. Previous studies are often based on peripheral whole-blood samples consisting of a collection of different cell types known to display very different DNA methylation profiles, and variability in cell-type composition has been demonstrated to lead to false-positive age-related associations of DNA methylation [22]. Hence, genome-wide DNA methylation profiles, and the extent and role of direct dynamic age-associated changes in DNA methylation during childhood, remain largely unexplored.

Here, we seek to identify intra-individual changes in DNA methylation during childhood through performing a comprehensive epigenome-wide association study (EWAS) using reduced representation bisulfite sequencing (RRBS). In order to investigate the role of cell-type composition (CTC) during aging, we applied a mediation analysis, which, in recent years, has been established within the framework of modern causal inference. Using this methodological framework, we were able to obtain information on the proportion of the age effect directly and indirectly through CTC on DNA methylation. To our knowledge, this is the first study investigating the dynamic nature of DNA methylation during childhood in longitudinal data dissecting the direct effect of aging from the effect mediated by cellular heterogeneity in blood samples.

## Results

### Data generation and quality assessment

We performed RRBS [23] in peripheral whole-blood derived DNA isolated from children (*n* = 19 individuals) at the age of 2, 10, and 16 years (*n* = 57 samples in total) selected from the prospective birth cohort “Environment and Childhood Asthma” (ECA) study [24]. Different methods were used for DNA extraction at the different time points, which could potentially introduce a bias. In order to investigate this, we ran a separate control experiment and compared the three methods in parallel using Infinium 450 K data from ten individuals. Overall, the different DNA extraction methods have little effect on DNA methylation (Additional file 1: Figure S1). Pairwise comparisons of the methods reveal small (<4 %) differences, of which none overlapped with the age-associated positions (aDMPs) identified in the present study (data not shown).

We sequenced an average of 28.9 million 75 bp single-end reads, 62.2 % aligned uniquely to the human genome. On average, 98.2 % of the aligned bases mapped to the 40–220 bp in silico-digested fragments. For some samples, a smaller fragment size range was excised from the gel, resulting in missing data. The total set of 1,902,432 CpGs with at least ten reads from at least one sample were filtered to only include autosomal CpGs from at least ten individuals with observations from all three time points, resulting in a final data set of 635,899 CpGs. Quality control of the DNA methylation values using principal component analysis (PCA) identified three outlier samples, which were excluded from the downstream analyses. All samples showed near complete (>99 %) bisulfite conversion of all non-CpG cytosines (data not shown).

### Widespread increase and decrease in DNA methylation is associated with age during childhood, with a varying proportion mediated by CTC variability

**Fig. 1:**
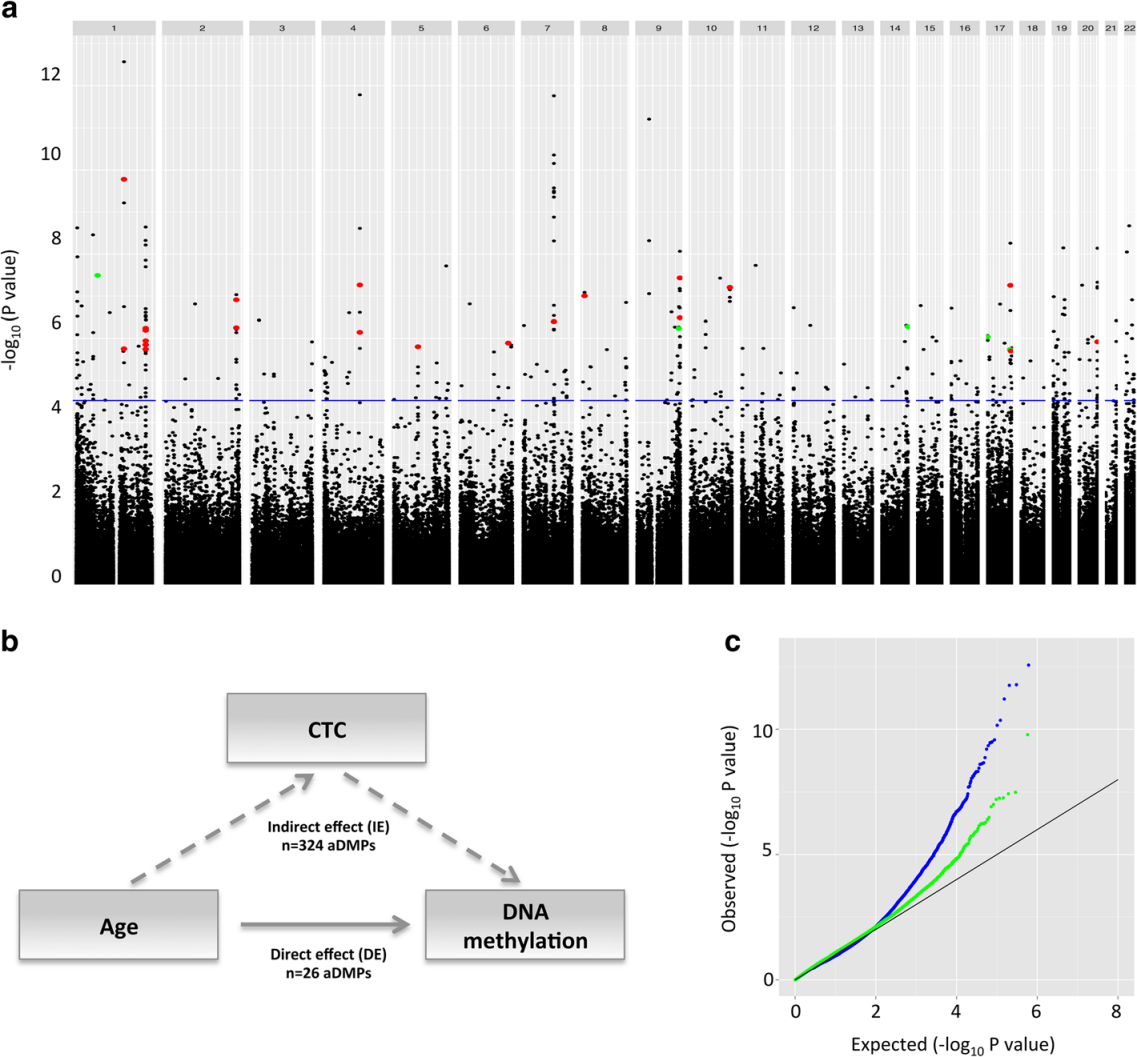
Age-dependent DNA methylation is partially mediated by CTC variability. **a** Manhattan plot of *p* values from the lme test. Each point represents a CpG (*n* = 635 899) with the chromosomal position along the *x*-axis and the negative logarithm of the associated *p* value on the *y*-axis. *Dashed line* represents the FDR (*blue*) at 5 %. The aDMPs significant after adjustment for CTC are marked in *red* (*n* = 21, which overlaps with the 346 CpGs) and *green* (*n* = 5, additional CpGs). **b** Illustration of the division of the total effect (TE) between age and DNA methylation in the direct effect (DE) illustrated by the *arrow* between age and DNA methylation and the indirect effect (ID) represented by the *dashed arrows* from age to CTC and from CTC to DNA methylation. **c** Quantile-quantile plot of the *p* values for tests of the TE between age and DNA methylation (in *blue*) and the DE between age and DNA methylation (in *green*). This plot indicates an enrichment of small *p* values for the test of the TE compared to the DE

To explore age-associated DNA methylation, we analyzed each CpG using a linear mixed-effects model (lme). The analysis of the total effect (TE) of age on DNA methylation identified 346 genome-wide significant age-associated differentially methylated positions (Fig. 1a) located in 178 genes. Of the 346 aDMPs, 196 (56.6 %) show a positive correlation and 150 (43.4 %) a negative correlation with age between 2 and 16 years as end points. The samples in this study consist of individuals with unambiguous asthma and non-asthmatic phenotypes, but we found no association between asthma and age-associated changes in DNA methylation (see the “Methods” section).

To assess whether differences in cell-type proportions (CTP) in peripheral whole-blood have an impact on the observed age-associated changes in DNA methylation, we investigated whether CTC acts (totally or partially) as a mediator. Following the rationale from modern causal inference theory (see the “Methods” section, Fig. 1b) in addition to the total effect (TE) presented above, we evaluated the direct effect (DE), the causal mediation effect, or the indirect effect (IE), as well as the proportion of the effect, which is acting through the CTC as a mediator. To do this, we used the available cell counts from five cell types (lymphocytes, neutrophils, monocytes, eosinophils, and basophils, Additional file 2: Figure S2).

Based on these analyses, 21 of the 346 aDMPs also show a genome-wide significant DE in addition to the significant TE. The distribution across all 346 aDMPs, with respect to the estimated proportion of the effect acting through the CTC as a mediator, is very widespread. Two hundred seventy-one of the 346 aDMPs were partially mediated with values between 0 and 1 (mean proportion mediated = 27 %, range = 2.7 to 67 %). Hence, the TE was stronger than the DE with an IE in the same direction compared to the TE and DE. Seventy-four out of 346 aDMPs had a negative proportion, meaning that the mediated effect direction was different from the direction of effect for both TE and DE (mean proportion mediated = −14 %, range = −1.3 to −41 %). In one case, the effect going through CTC as a mediator was stronger than the original effect. Interestingly, there appears to be a dependency between the degree of significance of the TE and the proportion of the effect going through CTC as a mediator. The standard deviation of CTC was 18 % for the lowest quartile of the aDMP *p* values and 40 % for the upper quartile, respectively (Additional file 3: Figure S3). Consequently, we conclude that the CTC variability across age has a varying impact on the age-associated DNA methylation in our study, and that this should be taken into account in the analyses.

After adjusting for CTC, we identified 26 genome-wide significant direct effect aDMPs associated with 12 genes (Table 1). Interestingly, one of the CpGs located in the promoter region of *TRIP6* was recently associated with pubertal transition in a recent EWAS [19]. Of these 26, 21 overlapped the total set of 346 aDMPs significant for TE. Hence, testing for the DE identified five additional aDMPs (Fig. 1a). The quantile-quantile plot of the *p* values corresponding to the tests for the TE and DE is shown in Fig. 1c, indicating an enrichment of small *p* values for TE compared to DE, supporting the concept of false-positive associations between age and DNA methylation due to different CTPs across age. This analysis strongly suggests that the effect of age on DNA methylation in whole blood is likely to be partially mediated by differences in CTPs. Consequently, based on these stringent assumptions, we chose to focus subsequent analyses on the 26 aDMPs which remain genome-wide significant after adjusting for varying CTC across age.

**Table 1:** Significant aDMPs with a direct effect on aging (*n* = 26)

Chr	Position	Adjusted *p* value	Mean difference 2 to 10 years	Mean difference 10 to 16 years	Mean difference 2 to 16 years	Gene	In CGI
1	69596098	0.0066	−0.0245	−0.0697	−0.0941	–	No
1	156883344^a^	0.0001	0.3168	0.0722	0.3891	PEAR1	No
1	156883372^a^	0.0463	0.1817	0.0111	0.1928	PEAR1	No
1	228400131	0.0263	0.2586	0.1004	0.3590	OBSNC	Yes
1	228400135	0.0401	0.1595	0.0644	0.2239	OBSNC	Yes
1	228400157	0.0463	0.2250	0.0287	0.2537	OBSNC	Yes
1	228400210	0.0271	0.1841	0.0520	0.2361	OBSNC	Yes
1	228400285	0.0427	0.2845	0.1512	0.4356	OBSNC	Yes
2	233251546	0.0263	0.3498	0.0427	0.3926	ECEL1P2	Yes
2	233251551	0.0097	0.3378	0.0274	0.3652	ECEL1P2	Yes
4	117279915	0.0066	−0.1557	−0.1123	−0.2681	–	No
4	117280012	0.0284	−0.2395	−0.0671	−0.3066	–	No
5	78985800^a^	0.0455	0.1730	0.1003	0.2732	CMYA5	No
6	158097037	0.0409	0.2276	0.0110	0.2386	–	No
7	100463812^a^	0.0254	−0.3432	−0.0831	−0.4262	TRIP6	Yes
8	6656321	0.0090	−0.1563	−0.0884	−0.2447	–	No
9	136474402	0.0263	0.0524	0.0942	0.1466	–	Yes
9	140173334	0.0066	0.2576	0.0925	0.3501	C9orf167	Yes
9	140173378	0.0229	0.0922	0.0555	0.1477	C9orf167	Yes
10	129537308	0.0066	0.3634	0.0584	0.4218	FOX12	Yes
14	105881000	0.0263	0.0370	0.0214	0.0584	–	No
17	1179934	0.0344	−0.1037	−0.1406	−0.2443	TUSC5	No
17	75421287	0.0463	0.0196	0.0271	0.0467	SEPT9	No
17	76355061^a^	0.0066	0.2076	0.1098	0.3174	SOCS3	Yes
17	78262132	0.0486	0.1670	0.0400	0.2069	RNF213	No
20	62679572^a^	0.0401	0.1111	0.0882	0.1993	SOX18	Yes

^a^Replicated CpG site

### Age-associated DNA methylation differences manifest early in childhood and are enriched for increased DNA methylation

**Fig. 2:**
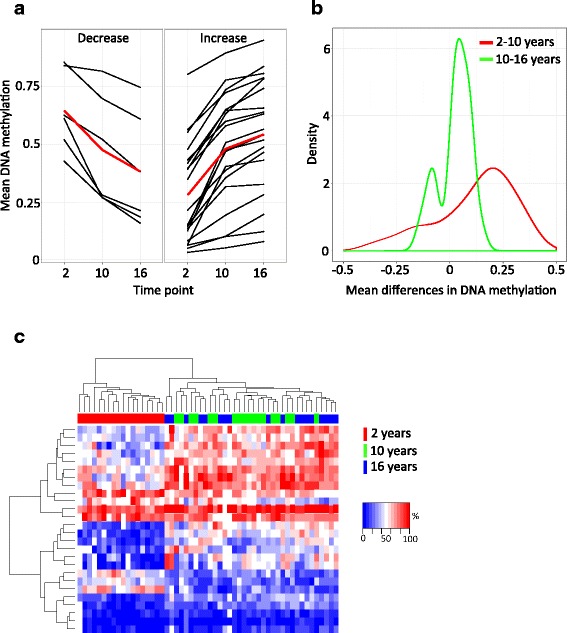
Mean DNA methylation differences between time points. **a** Line plots of mean DNA methylation on the *y*-axis against the time points on the *x*-axis separated by direction (decrease or increase in DNA methylation between 2 and 16 years as end points). **b** Density plots of the differences in mean DNA methylation between time points. **c** Heatmap of DNA methylation at aDMPs with rows representing aDMPs and columns representing samples. Cells are color scaled according to the level of DNA methylation (*blue* = low and *red* = high DNA methylation)

Of the 26 aDMPs with a direct effect on age-dependent DNA methylation, 20 showed an increase (76.9 %) and 6 (23.1 %) showed a decrease in mean DNA methylation across age with 2 and 16 years as end points (Fig. 2a). Hence, compared to the directionality of mean DNA methylation changes with a significant TE, adjustment for CTC results in a higher proportion of aDMPs showing an increase in DNA methylation. Fourteen (51.9 %) of the aDMPs are located in CpG islands that predominantly (92.9 %) show increased DNA methylation with age.

In order to investigate whether different periods during childhood are associated with different age-dependent changes in DNA methylation, we separated the two time points (2 to 10 years and 10 to 16 years) and explored the magnitude and direction of the age-associated changes in DNA methylation for the 26 aDMPs in more detail. The aDMPs display a wide range of DNA methylation, but the largest proportion exhibits intermediate DNA methylation (>25 and <75 % mean DNA methylation) across all time points (Fig. 2a). The majority of the aDMPs showed greater mean DNA methylation differences between 2 and 10 years (range = −0.34 to 0.36, median = 0.17) compared to 10 to 16 years (range = −0.14 to 0.15, median = 0.04) (Fig. 2b). Hence, the strongest age-associated DNA methylation differences are observed between 2 and 10 years. This observation is also supported by unsupervised hierarchical clustering of DNA methylation of the aDMPs, which clearly separates the 2-year olds from the 10- and 16-year olds and forms a discrete cluster (Fig. 2c).

For completeness, we also investigated the pairwise comparisons of the three time points at all CpGs with data from at least ten individuals at both time points with some variation. Results from these analyses revealed the same trend seen by the lme test with no additional CpGs showing age-associated changes in DNA methylation after adjustment for multiple testing.

### Age-associated DNA methylation is genome-wide and often shows regional clustering

**Fig. 3:**
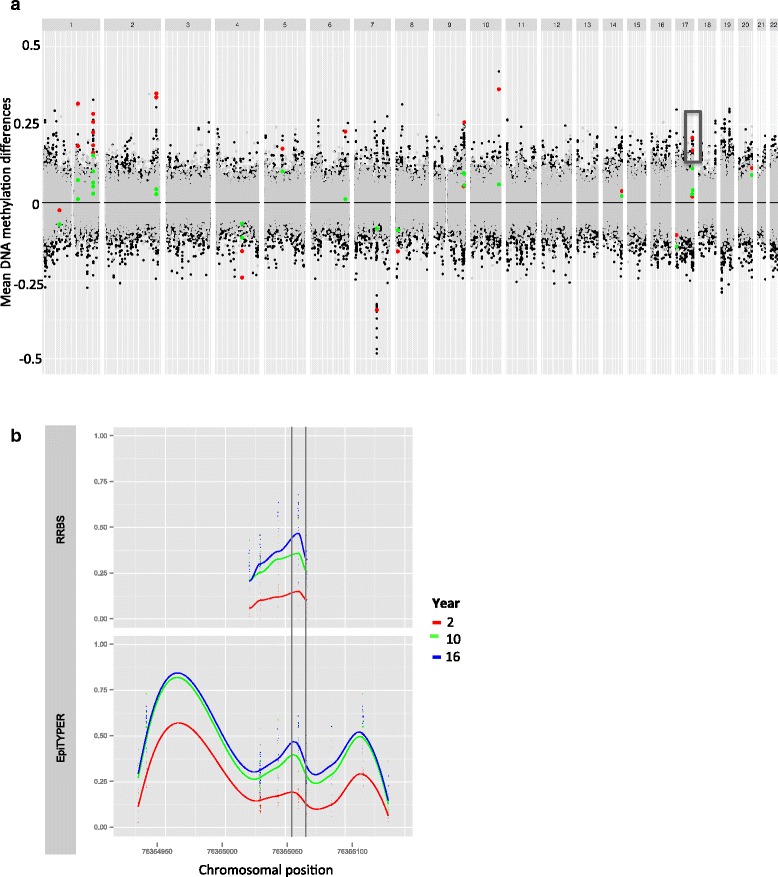
Chromosomal positions of mean DNA methylation differences. **a** Modified Manhattan plot of mean DNA differences by chromosomal position along the *x*-axis and mean DNA methylation differences on the *y*-axis. Each point represents a CpG site with mean DNA methylation differences between 2 and 10 years (*black*) and between 10 and 16 years (*gray*) for CpGs and mean DNA methylation differences between 2 and 10 years (*red*) and between 10 and 16 years (*green*) at the aDMPs (*n* = 26). **b** Scatter plot of DNA methylation at CpGs situated in a CpG island overlapping the promoter and the first exon of *SOCS3* generated by RRBS (*upper panel*) and EpiTYPER (replication data set, *bottom panel*) on the *y*-axis and chromosomal position on the *x*-axis. Each point represents a CpG per sample colored by age (*red* = 2, *blue* = 10, and *green* = 16 years) sorted by chromosomal position, and each point is jittered by 0.1). *Smoothening lines* represents local regression colored by time points. The significant aDMP (chr17: 76355061) is highlighted between vertical lines

CpGs are unevenly distributed in the genome but often form distinct regions and display considerable variation in DNA methylation [25]. Different regions in the genome are known to display differential DNA methylation (e.g., due to different biological functions as transcriptional control of associated genes). To explore the genomic localization of the significant aDMPs, we displayed the genomic location of the mean DNA methylation differences at the aDMPs per chromosome in a modified Manhattan plot (Fig. 3a). Although the differences in mean DNA methylation between the time points are distributed throughout the genome, some regions stand out and display large mean DNA methylation differences. For example, five neighboring CpGs located on chromosome 17 (chr17: 76355020–76355068) that are situated in a CpG island overlapping the promoter of *SOCS3* (suppressor of cytokine signaling 3) display a consistent large increase in mean DNA methylation between 2 and 10 years, whereas DNA methylation profiles at 10 and 16 years are indistinguishable (Fig. 3b, upper panel). Although there are distinct differences in DNA methylation between 2 and 10 years at all five CpGs, only one CpG showed genome-wide significance (marked between two vertical lines). This representative example demonstrates a limitation of this study related to the small sample size and illustrates that we are underpowered to detect the neighboring CpGs showing the same clear trend. The same tendency was also observed at CpGs adjacent to the majority of significant aDMPs located in CpG islands (results not shown).

### Replication

The Sequenom MassARRAY EpiTYPER platform was used to replicate a subset of the aDMPs in a larger set of samples (*n* = 31) selected from the same cohort. Using this approach, we were able to provide an independent measurement of DNA methylation at CpGs (*n* = 71) located in six genes showing age-associated DNA methylation (marked with an asterisk in Table 1). In situations where closely located CpGs could not be separated by cleavage as part of the protocol, the average DNA methylation measurement is presented (Additional file 4: Table S1). These data replicate the sequencing data and confirmed the age-associated DNA methylation identified by RRBS, and the 2-year olds separate from the 10- and 16-year olds (Fig. 3b and Additional file 5: Figure S4). Furthermore, as also observed with RRBS, the DNA methylation at aDMPs is a representative of neighboring CpGs in CpG islands.

### Age-associated loci have regulatory properties and developmental functions

Next, we aimed to extract biological meaning from the genes associated with age-associated DNA methylation. First, we used DAVID [26, 27] to explore the potential of shared biological terms and pathways. In line with the results presented above indicating that the study is underpowered (probably due to small sample size), we chose an exploratory approach and based the gene ontology (GO) analyses on genes associated with CpGs with a BH FDR < 25 % (*n* = 78 genes out of 15 339 genes included in the background list). Although a liberal cutoff, this captures a reasonable portion of the top ranked genes (0.5 % of the genes included) in the lme test. These GO analyses identified the enrichment of numerous relevant features with regulatory properties and developmental functions (Table 2). Specifically, the most significantly enriched categories have all regulatory functions with a potential role in different aspects of development.

**Table 2:** Biological features associated with aDMPs

GOTERM^a^	Description	Term	Count^b^	Frequency^c^	*p* value^d^
BP_1	Biological regulation	GO:0065007	36	46.2	0.0015
MF_4	Sequence-specific DNA binding	GO:0043565	11	14.1	0.0019
BP_2	Regulation of cellular process	GO:0050794	35	44.9	0.0023
MF_1	Transcription regulatory activity	GO:0030528	14	17.9	0.0028
BP_1	Developmental process	GO:0032502	20	25.6	0.0035
BP_2	Regulation of biological process	GO:0050789	35	44.9	0.0038
MF_FAT	DNA binding	GO:0003677	18	23.1	0.0046

^a^Resource where the term orient

^b^Number of genes in gene list (*n* = 152) which are involved in a specific GO term

^c^Percentage of the genes in input gene list which are involved in a specific GO term

^d^Adjusted according to Benjamini and Hochberg

## Discussion

This study has revealed intra-individual age-associated changes in DNA methylation across childhood, which is not mediated by variation in CTC in whole blood. In epigenetic studies based on whole-blood samples, the CTC is considered a major confounding factor due to the epigenetic heterogeneity of whole blood. For example, a declining number of a certain cell type across age in whole blood is likely to drive the observed age-associated change in DNA methylation. Consequently, the observed change in DNA methylation is simply due to a change in cell-type proportion rather than at the loci itself. We observed a consistent decrease and increase of lymphocyte and neutrophil cell counts, respectively, with age, in agreement with other studies identifying age-associated differences in blood cell counts [28] as well as estimated CTC in whole blood [22]. This is likely to influence the analyses and act as a mediator of the observed age-associated change in DNA methylation at sites also showing cell-type-specific DNA methylation in whole blood [29]. At these positions, it is not possible to dissect the effect coming from CTC variability alone from genuine age-associated changes in DNA methylation, which would also include cell-type-specific age-associated changes.

Several studies have identified tissue-specific age-associated DNA methylation [10, 21]. Although this has also been shown for cell types in whole blood [30], others have found that cell types explain a larger proportion of variability than age and that most sites show no significant association with age [22, 31], suggesting that the majority of age-associated DNA methylation is not cell-type specific in whole blood. However, this has mainly been explored in isolated CD4^+^ and CD14^+^ cells, accounting for most of the CTC, but blood is complex consisting of many cell types with diverse functions, each with unique potential roles in the aging process. Importantly, although genome-wide, this has mainly been investigated in a small fraction of the total CpGs in the human genome using Infinium 27K and 450K, leaving most of the CpGs unexplored in this context. Consequently, some of the age-associated changes in DNA methylation at the 324 CpGs mediated by CTC identified in our study using RRBS may potentially reflect cell-type-specific age-associated DNA methylation, although we do not consider this a common explanation.

The majority of the 26 aDMPs with a direct effect on aging are located in CpG islands associated with genes. Age-associated epigenetic changes are usually showing pronounced hypomethylation across the genome [8, 32]; however, the majority of aDMPs in our study involved increased DNA methylation. In particular, almost all significant aDMPs (and often the neighboring CpGs not reaching genome-wide significance) located in CpG islands are associated with increased DNA methylation. This is in agreement with previous studies and is believed to involve decreased gene expression of the nearby gene [15, 17, 33].

Several studies have now shown that DNA methylation changes with age at different genomic locations, the direction, and rate of change [9, 14, 33]. Our study shows a (generally) higher rate of change between 2 and 10 than between 10 and 16 years. We have not taken into account the differences in age range between the two time points, which in theory would have an impact and fit with the observation of epigenetic changes. However, we believe that the majority of the age-associated differences in DNA methylation associated with aging are probably not linear but involve controlled epigenetic changes at particular times during childhood.

The 26 aDMPs identified in our study overlapped 12 genes. Interestingly, *TRIP6*, which was hypomethylated between 2 and 16 years, was recently found to be associated with pubertal transition in another EWAS by Almstrup et al. [19]. This study is perhaps the most comparable to our study in terms of age range, tissue, and adjustment for differences in cell-type composition. Several factors involving variations in samples size, type of tissue, age range, and variations in cell-type composition not accounted for are likely to explain the lack of replication of any of the other genes previously shown to be associated with aging.

Ontology and pathway analyses revealed the enrichment of categories implicated in the development and regulation of biological processes, which are potentially important during different aspects of development during childhood. Typically, increased DNA methylation at CpG islands in proximity to genes is involved in transcriptional silencing. Differential DNA methylation levels close to genes involved in developmental processes have also been seen in other studies [10, 14, 15].

## Conclusions

The results presented in this study reveal positive and negative correlations of genome-wide changes in DNA methylation with age during childhood. Age-associated DNA methylation has regulatory roles on gene activity and developmental processes. The results reveal insights into the epigenetic dynamics associated with aging during childhood. Such information could ultimately point towards genomic regions and/or molecular pathways that are susceptible to aging-related disease-associated epigenetic dysregulation. The observed CTC variability in whole blood across age emphasizes the importance of assessing the total effect of CTC as a mediator. To our knowledge, this is the first study to survey DNA methylation dynamics during childhood in a longitudinal data set using RRBS.

## Methods

### Subjects and study design

The subjects in the present study were selected from the prospective birth cohort “Environment and Childhood Asthma” (ECA) study in Oslo [24]. The study includes the 19 children with available blood samples for methylation analyses at 2, 10, and 16 years of age (*n* = 57 samples in total) and with unambiguous clinical phenotypes (no asthma or allergy, consistent asthma and allergy, or crossovers between ages 2 to 16). Replication using EpiTYPER was performed in 31 additional individuals (*n* = 93 samples) selected from the same cohort.

### DNA extraction

From the 2-year-old subjects, DNA was extracted from blood clots obtained from the 2-year investigation (1993–1995) as described elsewhere [34]. From the 10-year-old subjects, DNA was extracted from peripheral whole-blood, using a MagnaPure LC (Roche). From the 16-year-old subjects, DNA was extracted from peripheral whole-blood on Autopure LS (Gentra/Qiagen) using the 2–5 × 10^7^ protocol.

### RNase and proteinaseK treatment of DNA

0.3–10 μg DNA was diluted in TE buffer (pH 8.0) to a final volume of 300 μl. RNase (USB) was added (final concentration 0.033 mg/mL), and the reactions were incubated at 37 °C for 30 min, followed by proteinaseK treatment (0.17 mg/mL) for 1 h at 55 °C. Purification was done using Genomic DNA Clean & Concentrator (Zymo Research) and eluted in 18 μl TE buffer.

### RRBS library preparation

Sequencing libraries were prepared based on two protocols, depending of the available amount of DNA.

#### 1 μg input

One microgram of RNase- and proteinaseK-treated DNA was digested with MspI (40U, New England Biolabs) at 37 °C for 2 h before purification (QIAquick Nucleotide Removal Kit, Qiagen) and eluted in 50 μl elution buffer (EB). End-repair was done using Illumina End Repair Mix with incubation for 30 min at 30 °C, followed by purification (QIAquick Nucleotide Removal Kit, Qiagen) and elution in 30 μl EB. The total volume of DNA was reduced to 15 μl (on a heating block), and DNA was adenylated using the Illumina A-tailing Mix (37 °C for 30 min). Illumina index-adapters (diluted 1:10 in dH_2_O) were ligated for 10 min at 30 °C, and the adapter-ligated fragments were purified (QIAquick PCR Purification Kit, Qiagen) and eluted in 30 μl EB. The samples were then separated on a 3 % NuSieve 3:1 agarose gel (Lonza). EtBr-stained gel slices containing adaptor-ligated fragments of 220–400 bp in size were excised and purified using two QIAquick MiniElute Kit (Qiagen) columns, with elution in a total of 20 μl EB. The adaptor-ligated and size-selected fragments were bisulfite-treated with the EpiTect Bisulphite Kit (Qiagen) using the formalin-fixed paraffin-embedded (FFPE) protocol and two consecutive rounds of 95 °C 5 min, 60 °C 25 min, 95 °C 5 min, 60 °C 85 min, 95 °C 5 min, and 60 °C 175 min and a final step at 20 °C 5 min and eluted in 20 μl EB. The final sequencing libraries were amplified in a 50-μl PCR containing 20 μl bisulfite-converted DNA, 14 μl dH_2_O, 5 μl 10 mM dNTPs, 5 μl 10× PfuTurbo Cx buffer, 5 μl TruSeq primer cocktail, and 1 μl PfuTurbo Cx hotstart DNA polymerase (2.5U, Agilent) at the following conditions: 95 °C for 5 min, followed by 17 cycles of 95 °C 20 s, 60 °C 30 s, 72 °C 30 s, and 72 °C for 7 min, and hold at 4 °C. The PCR reaction was purified using 90 μl of AMPure XP beads (Agencourt) and eluted in a final volume of 30 μl RSB buffer (Illumina) before quantified and analyzed using Qubit 2.0 (Invitrogen) and Bioanalyzer 2100 (Agilent), respectively. Libraries showing adapter dimers on the Bioanalyzer traces were subject to a second AMPure XP cleanup (DNA:bead ratio 1:1.25) and ran again on Qubit and Bioanalyzer. Finally, libraries were diluted to 10 nM in RSB buffer.

#### 150–400 ng input

One hundred fifty to four hundred nanograms of RNase- and proteinaseK-treated DNA was digested with MspI (20U, New England Biolabs) at 37 °C for 2 h before purification (QIAquick Nucleotide Removal Kit, Qiagen) and eluted in 50 μl EB. End repair was done using Illuminas End Repair Mix with incubation for 30 min at 30 °C, followed by purification (QIAquick Nucleotide Removal Kit, Qiagen). EB was diluted twice in nuclease-free water, and samples were eluted in 30 μl of this solution. The total volume of DNA was reduced to 15 μl (on a heating block), and DNA was adenylated using the Illumina A-tailing Mix (37 °C for 30 min). Illumina index-adapters (diluted 1:10 in dH_2_O) were ligated for 10 min at 30 °C, and the adapter-ligated fragments were purified (QIAquick PCR Purification Kit, Qiagen) and eluted in 30 μl EB. Each sample was mixed with 50 ng *Escherichia coli* carrier DNA (DNA prepared as described by Gu et al. [35]) and separated on a 3 % NuSieve 3:1 agarose gel (Lonza). EtBr-stained gel slices containing adaptor-ligated fragments of 220–400 bp in size were excised and purified on QIAquick MiniElute Kit (Qiagen), with an elution volume of 11 μl. The adaptor-ligated and size-selected fragments were bisulfite-treated with the EpiTect Bisulphite Kit (Qiagen) using the FFPE protocol and two consecutive rounds of 95 °C 5 min, 60 °C 25 min, 95 °C 5 min, 60 °C 85 min, 95 °C 5 min, and 60 °C 175 min and a final step at 20 °C 5 min and eluted in 20 μl EB. The bisulfite-converted DNA was mixed with 131 μl dH_2_O, 20 μl 10 mM dNTPs, 20 μl 10× PfuTurbo Cx buffer, 5 μl TruSeq primer cocktail, and 4 μl PfuTurbo Cx hotstart DNA polymerase (10U, Agilent). The mix was divided into eight aliquots of 25 μl in a 96-well PCR plate, and PCR was performed at the following conditions: 95 °C for 5 min, followed by 17 cycles of 95 °C 20 s, 60 °C 30 s, 72 °C 30 s, 72 °C for 7 min, and hold at 4 °C. The aliquots were pooled together, and the PCR was purified by AMPure XP beads (Agencourt) at DNA:bead ratio 1:1.25, eluted in a final volume of 30 μl RSB (Illumina), and quantified and analyzed using Qubit 2.0 (Invitrogen) and Bioanalyzer 2100 (Agilent), respectively. Libraries showing adapter dimers on the Bioanalyzer traces were subject to a second AMPure XP clean up (DNA/bead ratio 1:1.25) and ran again on Qubit and Bioanalyzer. Finally, libraries were diluted to 10 nM in RSB buffer (Illumina).

### Sequencing and alignment

In order to estimate an optimal input for library clustering, we performed a qPCR assay designed to amplify only those fragments carrying Illumina adapters at both ends. Inputs for clustering were calculated by comparing the amplification curves of our libraries to those of a previously sequenced RRBS library of known cluster density. For each subject, the three libraries (2, 10, and 16 years) were pooled according to calculated cluster input. Each pool was sequenced on one lane on a HiSeq 2000 (Illumina), generating 50 bp single-end reads. Raw sequencing data were processed by the standard Illumina pipeline for image analysis and base calling. Quality evaluation of raw sequence data was generated using FastQC (http://www.bioinformatics.bbsrc.ac.uk/projects/fastqc). Genomic alignment was performed by RRBSMAP [36] (v1.6). The genome (hg19) was indexed on CCGG (MspI restriction site), and a maximum of two mismatches on a read allowed and 3′-end adapter sequences were trimmed off to optimize the alignment of short MspI fragments. Alignment efficiency was calculated using the Picard CalculateHsMetrics tool (http://broadinstitute.github.io/picard). A minimum of ten reads was used to calculate the DNA methylation at C in a CpG context. The DNA methylation level at a CpG was extracted from the mapping results as the number of methylated Cs divided by the total number of Cs using the python script included in RRBSMAP. The bisulfite conversion rate was estimated based on the frequency of C-to-T conversions for cytosines that were not in a CpG context. For biological interpretation, CpGs were annotated by using the annotation module in SAAP-RRBS [37] and BEDTools [38].

### Sequenom MassARRAY replication

Replication of age-associated DNA methylation in six genes covered by 71 CpGs was performed using the Sequenom MassARRAY EpiTYPER (Sequenom Inc, Hamburg, Germany). PCR primers (Additional file 6: Table S2) were designed using the Sequenom EpiDesigner software (www.epidesigner.com). To reduce variability in DNA methylation resulting from technical variation during PCR, each sample was amplified in triplicate and pooled prior to the MassARRAY analysis.

### Statistical analysis

All statistical tests were conducted in R (www.r-project.org).

#### Analysis of age-associated DNA methylation

To investigate the total effect (TE) of age on DNA methylation in whole blood, we used a linear mixed-effects model with time as a fixed and subjects as a random factor. This model is suitable for repeated measurements and implemented in the *lme4* and *lmeTest* R packages, which are able to deal with missing values. This is an advantage for RRBS datasets in particular, since it is challenging to produce a completely overlapping data set from all samples. Statistical analyses were performed for each CpG with data from at least ten individuals. Pairwise tests were done for all CpGs with at least ten complete pairs (2 to 10, 10 to 16, and 2 to 16 years). Adjustment for multiple testing was performed, considering a false discovery rate (FDR) below 5 % to be genome-wide significant using the method of Benjamini and Hochberg [39].

#### Analysis of cell-type proportions as a mediator

Cell counts for five cell types were available (lymphocytes, neutrophils, monocytes, eosinophils, and basophils) for all individuals and time points. The cell-type proportions (CTPs) were calculated by dividing the cell counts for a certain cell type by the sum for the cell counts across all cell types. The lymphocyte CTPs were not available for 2-year olds and were imputed by calculating the difference between 100 % and the four remaining CTPs. In addition, there were eight missing values across all cell types at time point 10 years and one at time point 16 years. Here, missing values were imputed by taking the mean within each cell type and time point. As can be seen in Figure S2 (Additional file 2), lymphocytes and neutrophils together explain on average 89 % of all CTPs. Using principal component analysis (*irlba* package in R) on the CTP data matrix, the first principal component (PC1) was evaluated. The two main cell types are well represented by PC1 with loadings of 98 and 96 %, respectively. The remaining three cell types were neglected because the possible impact of potential cell-type-specific DNA methylation changes would not be captured by DNA methylation values based on whole blood anyway. As an example, a change of 50 % within a cell type with a proportion of 5 % would lead to an overall DNA methylation change of 0.5 × 0.05 = 2.5 % given that DNA methylation in the other cell types remains constant. Thus, we use PC1 when investigating the role of CTPs in our analysis. In order to find out whether CTC acts (totally or partially) as a mediator, modern causal inference theory has been used to estimate different types of effects [40]. The analysis of mixed-models modeling CTPs as a mediator was done by using the *mediation* R package [41]. The results were corrected for multiple testing by the method of Benjamini and Hochberg [39] and considered genome-wide significant on the 5 % level (FDR).

#### GO analyses

GO analysis was performed using the DAVID functional annotation tool [26, 27] based on the results of the initial association tests for age and DNA methylation adjusted for CTC. The CpGs included were represented by 16,687 genes, of which 15,339 genes have a DAVID id. Out of these, 87 genes (78 with a DAVID id) had an adjusted *p* value ≤0.25. The analysis was done with default parameters, and the results were corrected for multiple testing by the method of Benjamini and Hochberg [39].

### Investigation of asthma disease status as a confounder

In order to investigate whether the asthma phenotype was confounding the analyses of age-associated DNA methylation, we tested the representativeness of the control samples (with no asthma diagnosis) for the whole sample for all significant aDMPs identified in our study. We used a sampling procedure for investigating the DNA methylation differences between controls and the whole sample. For each time point and significant aDMP, we calculate the mean DNA methylation (%) difference between the control group (eight samples) and the whole group (19 samples). In addition, we derive the empirical distribution for these mean differences (selection versus all) for all possible combinations (eight out of 19). Given this distribution, we can evaluate for each time point whether the number of probes with “extreme differences” in DNA methylation for the control group compared to the overall sample is higher than expected. Overall, there are no larger differences in mean DNA methylation between the control group and the overall group than expected by chance. For example, the positive rates on the 5 % significance level across all aDMPs are 4.3 % at 2 years and 1.4 % at both 10 and 16 years, which is (much) less than expected. In addition, testing the significant aDMPs for an association with asthma revealed no significant results either (results not shown).

## Additional files

Additional file 1: Figure S1. MDS plot of extraction methods. Multidimensional scaling plots of the top 1000 most variable positions between ten samples isolated with three different extraction methods (A = AutoPure LS, M = MagNA Pure, O = organic). The first MDS component is on the *x*-axis, and the second MDS component on the *y*-axis. (PDF 255 kb)
Additional file 2: Figure S2. Mean cell-type proportions including 95 % confidence intervals at age 2, 10, and 16 years. (PNG 433 kb)
Additional file 3: Figure S3. Scatterplot of the –log (TE) *p* values of the 346 age-associated differentially methylated positions (aDMPs) versus the mean proportion mediated by cell-type proportions. Standard deviation of CTC for the lowest quartile of the *p* values of these aDMPs was 18 % and for the upper quartile 40 %, respectively. (PNG 138 kb)
Additional file 4: Table S1. EpiTYPER results. (XLSX 31 kb)
Additional file 5: Figure S4. Heatmap of DNA methylation measured by EpiTYPER at CpGs (*n* = 71) with rows representing CpGs and columns representing samples. Cells are color scaled according to the level of DNA methylation (blue = low and red = high DNA methylation). (PDF 744 kb)
Additional file 6: Table S2. EpiTYPER primers. (XLS 26 kb)

## Abbreviations

aDMPs: Age-associated differentially methylated positions
BH: Benjamini and Hochberg
CTC: Cell-type composition
CTP: Cell-type proportion
DE: Direct effect
ECA: Environmental and childhood asthma study
EWAS: Epigenome-wide association study
FDR: False discovery rate
Gb: Giga bases
IE: Indirect effect
lme: Linear mixed-effects model
PCA: Principal component analysis
RRBS: Reduced representation bisulfite sequencing
TE: Total effect

## References

[1] Ji H, Ehrlich LIR, Seita J, Murakami P, Doi A, Lindau P (2010). Comprehensive methylome map of lineage commitment from haematopoietic progenitors. Nature.

[2] Baran Y, Subramaniam M, Biton A, Tukiainen T, Tsang EK, Rivas MA (2015). The landscape of genomic imprinting across diverse adult human tissues. Genome Res.

[3] Wagner JR, Busche S, Ge B, Kwan T, Pastinen T, Blanchette M (2014). The relationship between DNA methylation, genetic and expression inter-individual variation in untransformed human fibroblasts. Genome Biol.

[4] Varley KE, Gertz J, Bowling KM, Parker SL, Reddy TE, Pauli-Behn F (2013). Dynamic DNA methylation across diverse human cell lines and tissues. Genome Res.

[5] Esteller M (2008). Epigenetics in cancer. N. Engl. J. Med..

[6] Javierre BM, Fernandez AF, Richter J, Al-Shahrour F, Martin-Subero JI, Rodriguez-Ubreva J (2010). Changes in the pattern of DNA methylation associate with twin discordance in systemic lupus erythematosus. Genome Res.

[7] Dick KJ, Nelson CP, Tsaprouni L, Sandling JK, Aïssi D, Wahl S (2014). DNA methylation and body-mass index: a genome-wide analysis. Lancet.

[8] Teschendorff AE, West J, Beck S (2013). Age-associated epigenetic drift: implications, and a case of epigenetic thrift?. Hum Mol Genet.

[9] Jones MJ, Goodman SJ, Kobor MS (2015). DNA methylation and healthy human aging. Aging Cell.

[10] Horvath S, Zhang Y, Langfelder P, Kahn RS, Boks MPM, van Eijk K (2012). Aging effects on DNA methylation modules in human brain and blood tissue. Genome Biol.

[11] Kananen L, Marttila S, Nevalainen T, Kummola L, Junttila I, Mononen N (2016). The trajectory of the blood DNA methylome ageing rate is largely set before adulthood: evidence from two longitudinal studies. Age.

[12] Levine ME, Lu AT, Chen BH, Hernandez DG, Singleton AB (2016). Menopause accelerates biological aging. PNAS.

[13] Teschendorff AE, Menon U, Gentry-Maharaj A, Ramus SJ, Weisenberger DJ, Shen H (2010). Age-dependent DNA methylation of genes that are suppressed in stem cells is a hallmark of cancer. Genome Res.

[14] Alisch RS, Barwick BG, Chopra P, Myrick LK, Satten GA, Conneely KN (2012). Age-associated DNA methylation in pediatric populations. Genome Res.

[15] Bell JT, Tsai P-C, Yang T-P, Pidsley R, Nisbet J, Glass D (2012). Epigenome-wide scans identify differentially methylated regions for age and age-related phenotypes in a healthy ageing population. PLoS Genet.

[16] Martino DJ, Tulic MK, Gordon L, Hodder M, Richman TR, Metcalfe J (2014). Evidence for age-related and individual-specific changes in DNA methylation profile of mononuclear cells during early immune development in humans. Epigenetics.

[17] Acevedo N, Reinius LE, Vitezic M, Fortino V, Söderhäll C, Honkanen H (2015). Age-associated DNA methylation changes in immune genes, histone modifiers and chromatin remodeling factors within 5 years after birth in human blood leukocytes. Clin Epigenetics.

[18] Urdinguio RG, Torró MI, Bayón GF, Alvarez-Pitti J, Fernandez AF, Redon P (2016). Longitudinal study of DNA methylation during the first 5 years of life. J Transl Med.

[19] Almstrup K, Johansen ML, Busch AS, Hagen CP, Nielsen JE, Petersen JH (2016). Pubertal development in healthy children is mirrored by DNA methylation patterns in peripheral blood. Sci Rep.

[20] Maegawa S, Hinkal G, Kim HS, Shen L, Zhang L, Zhang J (2010). Widespread and tissue specific age-related DNA methylation changes in mice. Genome Res.

[21] Day K, Waite LL, Thalacker-Mercer A, West A, Bamman MM, Brooks JD (2013). Differential DNA methylation with age displays both common and dynamic features across human tissues that are influenced by CpG landscape. Genome Biol.

[22] Jaffe AE, Irizarry RA (2014). Accounting for cellular heterogeneity is critical in epigenome-wide association studies. Genome Biol.

[23] Meissner A (2005). Reduced representation bisulfite sequencing for comparative high-resolution DNA methylation analysis. Nucleic Acids Res.

[24] Lødrup Carlsen KC (2002). The environment and childhood asthma (ECA) study in Oslo: ECA-1 and ECA-2. Pediatr Allergy Immunol Munksgaard.

[25] Shen H, Qiu C, Li J, Tian Q, Deng H-W (2013). Characterization of the DNA methylome and its interindividual variation in human peripheral blood monocytes. Epigenomics.

[26] Huang DW, Sherman BT, Lempicki RA (2009). Bioinformatics enrichment tools: paths toward the comprehensive functional analysis of large gene lists. Nucleic Acids Res.

[27] Huang DW, Sherman BT, Lempicki RA (2008). Systematic and integrative analysis of large gene lists using DAVID bioinformatics resources. Nat Protoc.

[28] Mahlknecht U, Kaiser S (2010). Age-related changes in peripheral blood counts in humans. Exp Ther Med.

[29] Liang L, Cookson WOC (2014). Grasping nettles: cellular heterogeneity and other confounders in epigenome-wide association studies. Hum Mol Genet.

[30] Chu M, Siegmund KD, Hao Q-L, Crooks GM, Tavaré S, Shibata D (2008). Inferring relative numbers of human leucocyte genome replications. Br J Haematol.

[31] Rakyan VK, Down TA, Maslau S, Andrew T, Yang TP, Beyan H (2010). Human aging-associated DNA hypermethylation occurs preferentially at bivalent chromatin domains. Genome Res.

[32] Heyn H, Li N, Ferreira HJ, Moran S, Pisano DG, Gomez A (2012). Distinct DNA methylomes of newborns and centenarians. Proc. Natl. Acad. Sci. U.S.A.

[33] McClay JL, Aberg KA, Clark SL, Nerella S, Kumar G, Xie LY (2014). A methylome-wide study of aging using massively parallel sequencing of the methyl-CpG-enriched genomic fraction from blood in over 700 subjects. Hum Mol Genet.

[34] Munthe-Kaas MC, Torjussen TM, Gervin K, Lødrup Carlsen KC, Carlsen KH, Granum B (2010). CD14 polymorphisms and serum CD14 levels through childhood: a role for gene methylation?. J Allergy Clin Immunol.

[35] Gu H, Smith ZD, Bock C, Boyle P, Gnirke A, Meissner A (2011). Preparation of reduced representation bisulfite sequencing libraries for genome-scale DNA methylation profiling. Nat Protoc.

[36] Xi Y, Bock C, Muller F, Sun D, Meissner A, Li W (2012). RRBSMAP: a fast, accurate and user-friendly alignment tool for reduced representation bisulfite sequencing. Bioinformatics.

[37] Sun Z, Baheti S, Middha S, Kanwar R, Zhang Y, Li X (2012). SAAP-RRBS: streamlined analysis and annotation pipeline for reduced representation bisulfite sequencing. Bioinformatics.

[38] Quinlan AR, Hall IM (2010). BEDTools: a flexible suite of utilities for comparing genomic features. Bioinformatics.

[39] Benjamini Y (2010). Discovering the false discovery rate. J R Stat Soc Series B Stat Methodology.

[40] Imai K, Keele L, Tingley D (2010). A general approach to causal mediation analysis. Psychol Methods.

[41] Tingley D, Yamamoto T, Hirose K, Keele L, Imai K. Mediation: R package for causal mediation analysis. 2014;59:1–34.

