# Supplementary figures and images for: Intra-individual changes in DNA methylation not mediated by cell-type composition are correlated with aging during childhood
Source: Clin Epigenetics. 2016 Oct 21;8:110. doi: 10.1186/s13148-016-0277-3 (PMC5073885; doi:10.1186/s13148-016-0277-3)

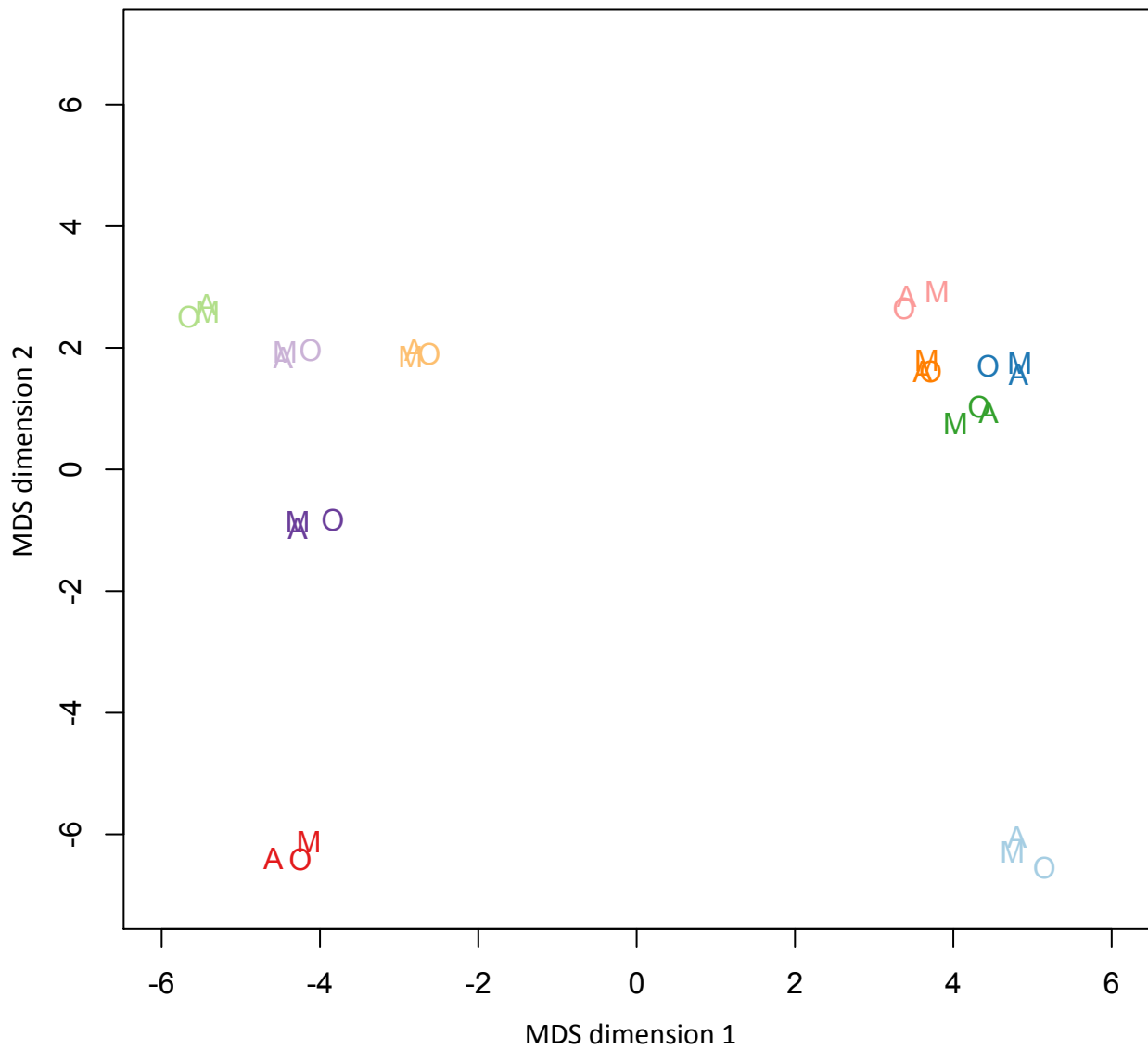

Supplement: Additional file 1: Figure S1. — MDS plot of extraction methods. Multidimensional scaling plots of the top 1000 most variable positions between ten samples isolated with three different extraction methods (A = AutoPure LS, M = MagNA Pure, O = organic). The first MDS component is on the x-axis, and the second MDS component on the y-axis. (PDF 255 kb) [file 13148_2016_277_MOESM1_ESM.pdf]

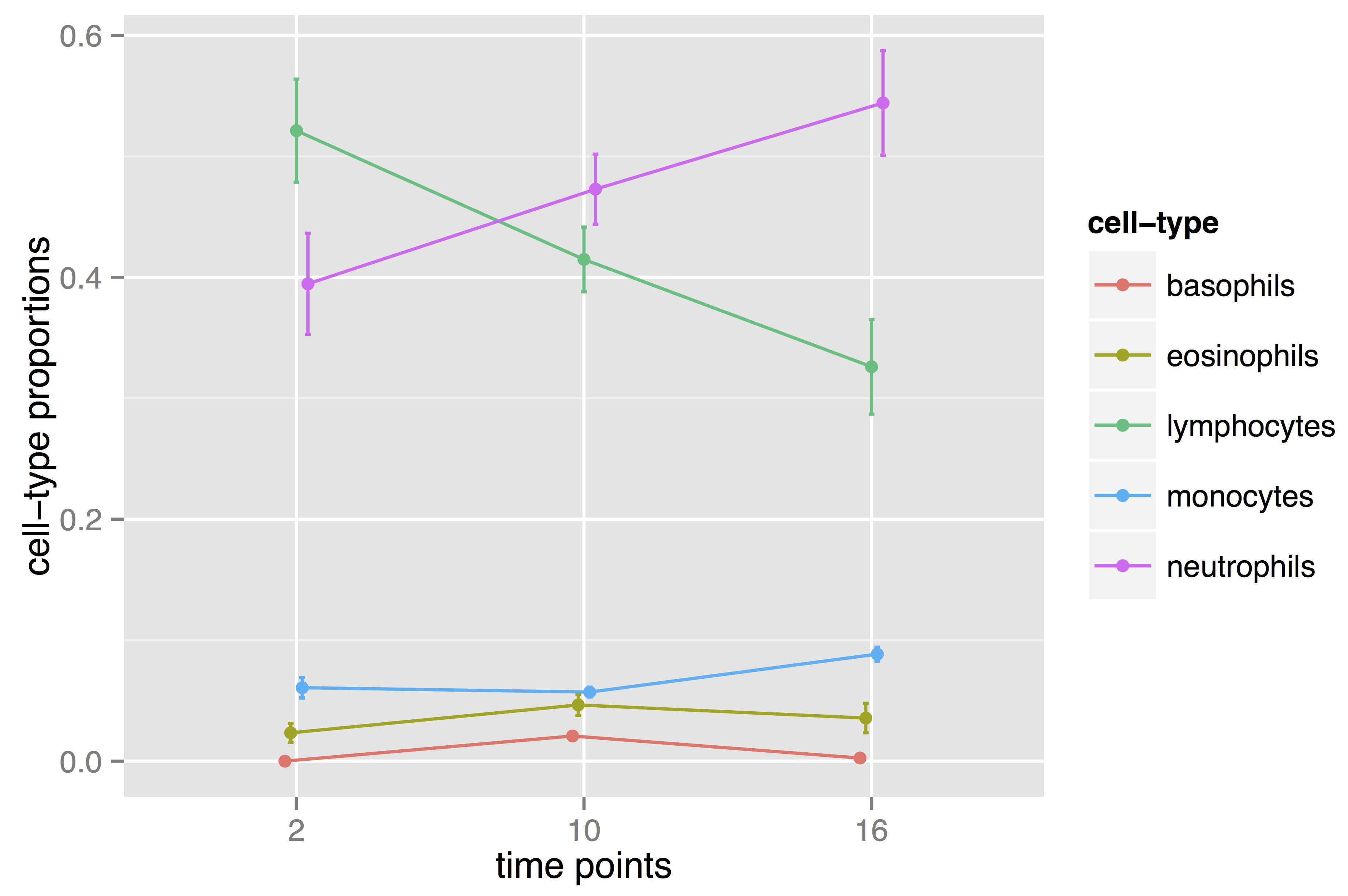

Supplement: Additional file 2: Figure S2. — Mean cell-type proportions including 95 % confidence intervals at age 2, 10, and 16 years. (PNG 433 kb) [file 13148_2016_277_MOESM2_ESM.png]

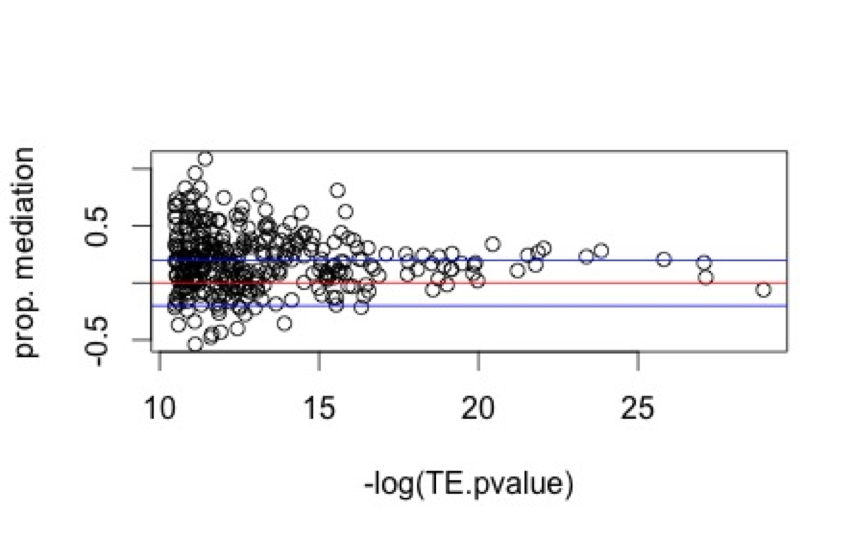

Supplement: Additional file 3: Figure S3. — Scatterplot of the –log (TE) p values of the 346 age-associated differentially methylated positions (aDMPs) versus the mean proportion mediated by cell-type proportions. Standard deviation of CTC for the lowest quartile of the p values of these aDMPs was 18 % and for the upper quartile 40 %, respectively. (PNG 138 kb) [file 13148_2016_277_MOESM3_ESM.png]

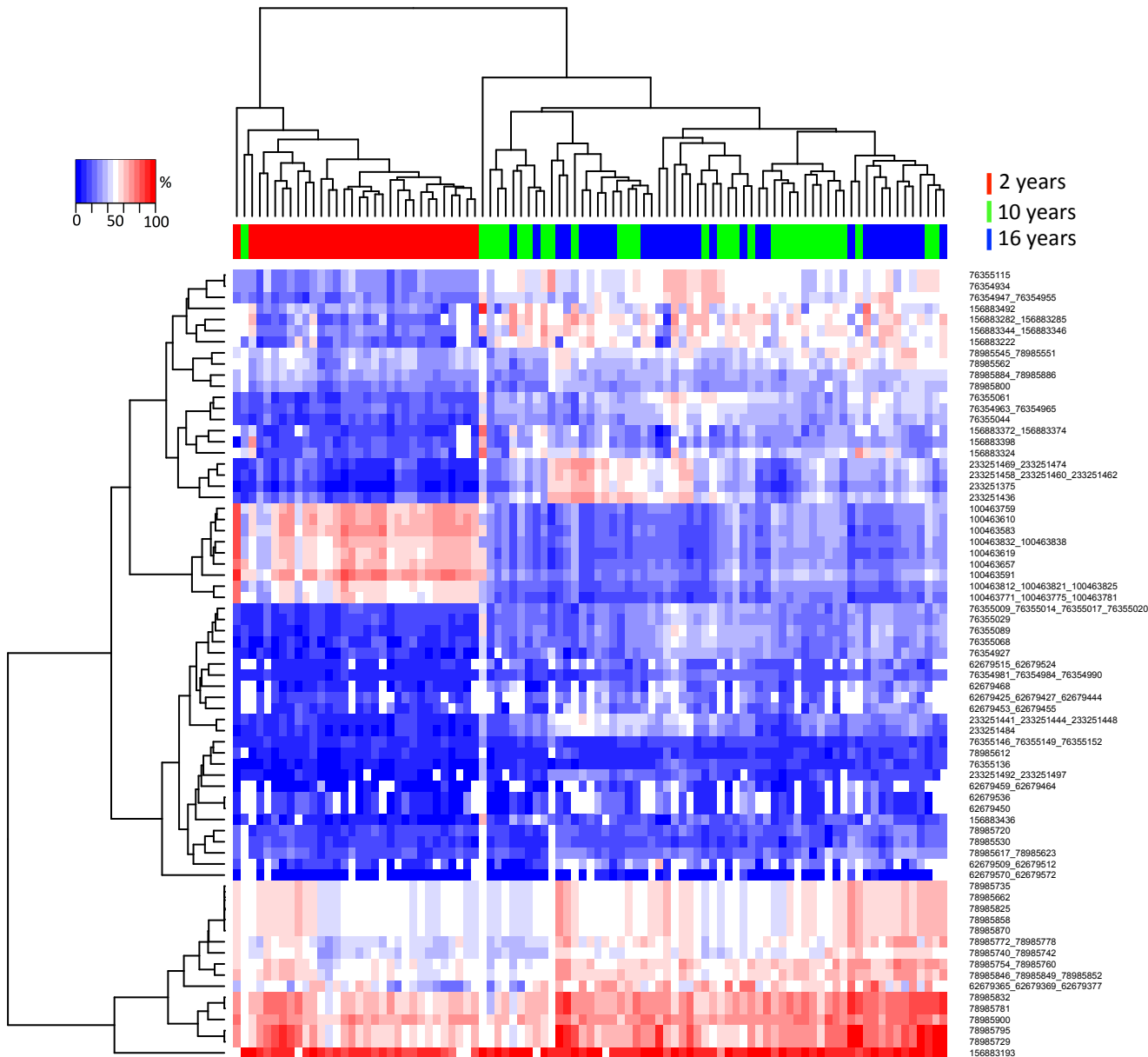

Supplement: Additional file 5: Figure S4. — Heatmap of DNA methylation measured by EpiTYPER at CpGs (n = 71) with rows representing CpGs and columns representing samples. Cells are color scaled according to the level of DNA methylation (blue = low and red = high DNA methylation). (PDF 744 kb) [file 13148_2016_277_MOESM5_ESM.pdf]
